# MicroRNA-155 Modulates Treg and Th17 Cells Differentiation and Th17 Cell Function by Targeting SOCS1

**DOI:** 10.1371/journal.pone.0046082

**Published:** 2012-10-16

**Authors:** Rui Yao, Yu-Lan Ma, Wei Liang, Huan-Huan Li, Zhi-Jun Ma, Xian Yu, Yu-Hua Liao

**Affiliations:** 1 Laboratory of Cardiovascular Immunology, Institute of Cardiology, Union Hospital, Huazhong University of Science and Technology, Wuhan, Hubei, China; 2 Department of General Surgery, Union Hospital, Huazhong University of Science and Technology, Wuhan, Hubei, China; International Center for Genetic Engineering and Biotechnology, India

## Abstract

MicroRNA (miR)-155 is a critical player in both innate and adaptive immune responses. It can influence CD4^+^ T cell lineage choice. To clarify the role of miR-155 in CD4^+^ CD25^+^ regulatory T (Treg)/T helper (Th)17 cell differentiation and function, as well as the mechanism involved, we performed gain-and loss-of-function analysis by transfection pre-miR-155 and anti-miR-155 into purified CD4^+^ T cells. The results showed that miR-155 positively regulated both Treg and Th17 cell differentiation. It also induced the release of interleukin (IL)-17A by Th17 cells, but not the release of IL-10 and transforming growth factor (TGF)-β1 by Treg cells. Furthermore, we found that miR-155 reacted through regulating Janus kinase/signal transducer and activator of transcription (JAK/STAT) rather than TGF-β/mothers against decapentaplegic homolog (SMAD) signaling pathway in the process of Treg and Th17 cells differentiation. This may because suppressors of cytokine signaling (SOCS)1, the important negative regulator of JAK/STAT signaling pathway, was the direct target of miR-155 in this process, but SMAD2 and SMAD5 were not. Therefore, we demonstrated that miR-155 enhanced Treg and Th17 cells differentiation and IL-17A production by targeting SOCS1.

## Introduction

A novel small non-coding RNA species known as microRNAs (miRNAs, miRs) is involved in biological control at multiple levels. They regulate gene expression essential for cell development and function through mRNA degradation or translational inhibition. Evidences indicated that miRNAs played important roles in immune system. In T lymphocytes, conditional deletion of Dicer, a RNase III enzyme which is critical for the generation of mature miRNAs, resulted in impaired thymic development and diminished T helper (Th) cell differentiation [1], [2]. And Dicer deficiency in B lymphocytes showed a block in B lymphocytes development [3]. Subsequent reports revealed the roles of individual miRNAs (such as miR-181, miR-146a and miR-155) in immune system. MiR-181 induced B lymphocytes differentiation from hematopoietic stem cells [4]. MiR-146a negatively regulated innate immune response by interfering Toll-like receptor (TLR) signaling pathway [5], [6]. MiR-155 has been found apparently up-regulated in several activated immune cells, including T lymphocytes, B lymphocytes, macrophages and dendritic cells (DCs). It is up-regulated by a broad range of inflammatory mediators (such as tumor necrosis factor-α, lipopolysaccharide, and polyriboinosinic: polyribocytidylic acid, et al.) during innate immune response [7], [8]. Thai et al. [9] demonstrated that *bic*/miR-155^−/−^ mice showed diminished geminal center response. While Rodriguez et al. [10] found that *bic*/miR-155^−/−^ CD4^+^ T cells were intrinsically biased toward Th2 cell differentiation rather than Th1 cell. These studies indicated that miR-155 involved in both innate and adaptive immune responses.

CD4^+^ CD25^+^ regulatory T (Treg) and Th17 cells are novel Th cell subsets distincted from Th1 and Th2 cells. Treg is a suppressive Th cell subset. It functions in maintenance of self-tolerance and preventing the development of various inflammatory diseases by directly contacting effective immune cells and secreting anti-inflammatory cytokines, like interleukin (IL)-10 and transforming growth factor (TGF)-β1 [11]. Th17 is a pro-inflammatory Th cell subset. It plays important roles in host defense and is involved in various autoimmune and inflammatory diseases mainly by secreting IL-17A and other cytokines, like IL-21 and IL-22 [12]. Recently, Kohlhaas et al. [13] and Lu et al. [14] found that miR-155 was required for Treg development but was dispensable for the function of Treg in regulating conventional T cells. O'Connell et al. [15] demonstrated that miR-155 was necessary for Th17 cell differentiation, and miR-155 deficient mice failed to develop into experimental autoimmune encephalomyelitis (EAE) when treated with myelin oligodendrocyte glycoprotein (MOG). However, the way miR-155 regulating Treg and Th17 cells differentiation and their functional cytokines production, as well as the precise mechanisms involved are still unknown.

In the present study, we detected the roles of miR-155 in Treg and Th17 cells differentiation and cytokines production by over-expression and inhibition of miR-155. And we also confirmed the direct target of miR-155 that involved.

## Results

### MiR-155 regulates the differentiation of Treg and Th17 cells and the expression of Foxp3 and RORγt

**Figure 1:**
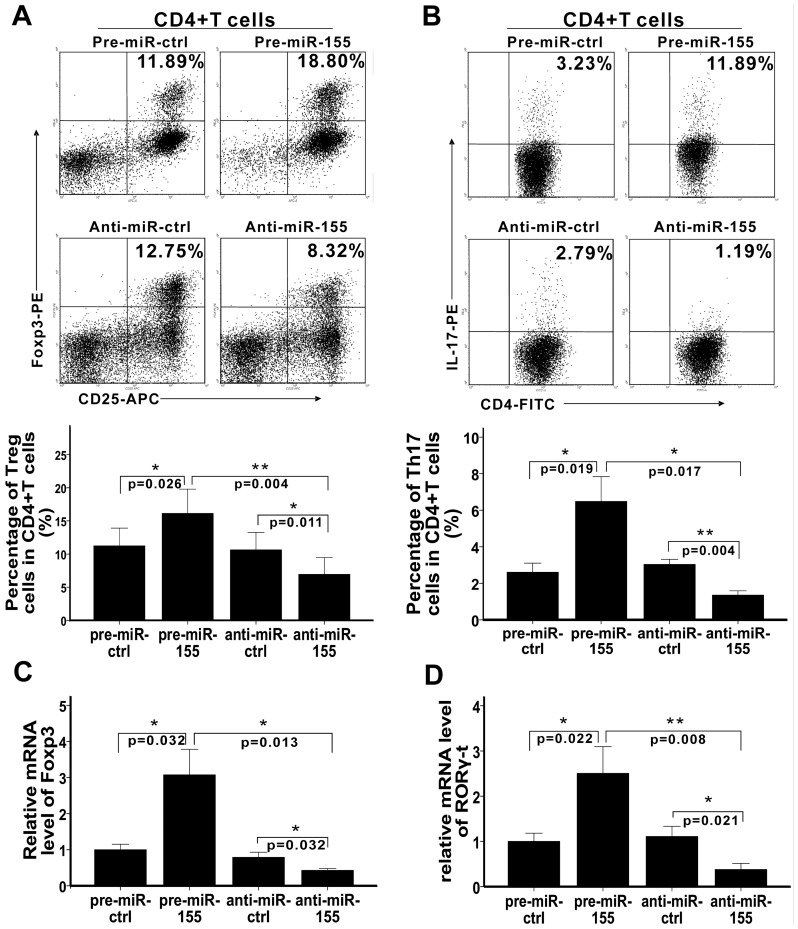
MiR-155 regulates Treg and Th17 cells differentiation as well as Foxp3 and RORγt expression. Pre-miR-ctrl, pre-miR-155, anti-miR-ctrl, and anti-miR-155 were transfected into CD4^+^ T cells, which were then activated and polarized. A and B. The frequencies of Treg and Th17 cells in CD4^+^ T cells were determined by flow cytometry 4 days later. Treg (A) and Th17 (B) cells were gated with CD4^+^CD25^+^Foxp3^+^ and CD4^+^IL-17^+^, respectively. Representative FACS pictures from a single case are shown. And the percentages of positive cells in CD4^+^ T cells are shown in each panel. The collective results of three independent experiments are shown in the histograms as mean ± SD. C and D. The mRNA levels of Foxp3 (C) and RORγ-t (D) were detected by RT-PCR 3 days after transfection and activation. Relative expression of them were collectively analysed and the results are shown as mean ± SD. Data represent three independent experiments. *p<0.05, **p<0.01.

To explore the roles of miR-155 in Treg and Th17 cells differentiation, miR-155 was over-expressed and inhibited by pre-miR-155 and anti-miR-155, respectively. The results showed that both the frequencies of Treg and Th17 cells were higher in CD4^+^ T cells which were transfected with pre-miR-155 than in those which were transfected with pre-miR-ctrl and anti-miR-155 (Fig. 1A, 1B). In contrast, both of them were lower in CD4^+^ T cells which were transfected with anti-miR-155 than in those which were transfected with anti-miR-ctrl (Fig. 1A, 1B). Forkhead/winged helix transcription factor (Foxp3) and retinoic acid-related orphan receptor γt (RORγt) are important transcriptional regulators that regulate Treg and Th17 cells differentiation and function. So, we also detected the mRNA levels of Foxp3 and RORγt when miR-155 was over-expressed and inhibited. Similarly, both Foxp3 and RORγt expression were increased in pre-miR-155 groups compared with those in pre-miR-ctrl groups and anti-miR-155 groups and were decreased in anti-miR-155 groups compared with those in anti-miR-ctrl groups (Fig. 1C, 1D). So, the results reveal that miR-155 positively regulates Treg and Th17 cells differentiation as well as Foxp3 and RORγt expression.

### MiR-155 regulates the secreting of IL-17A by Th17 cells but not the secreting of IL-10 and TGF-β1 by Treg cells

**Figure 2:**
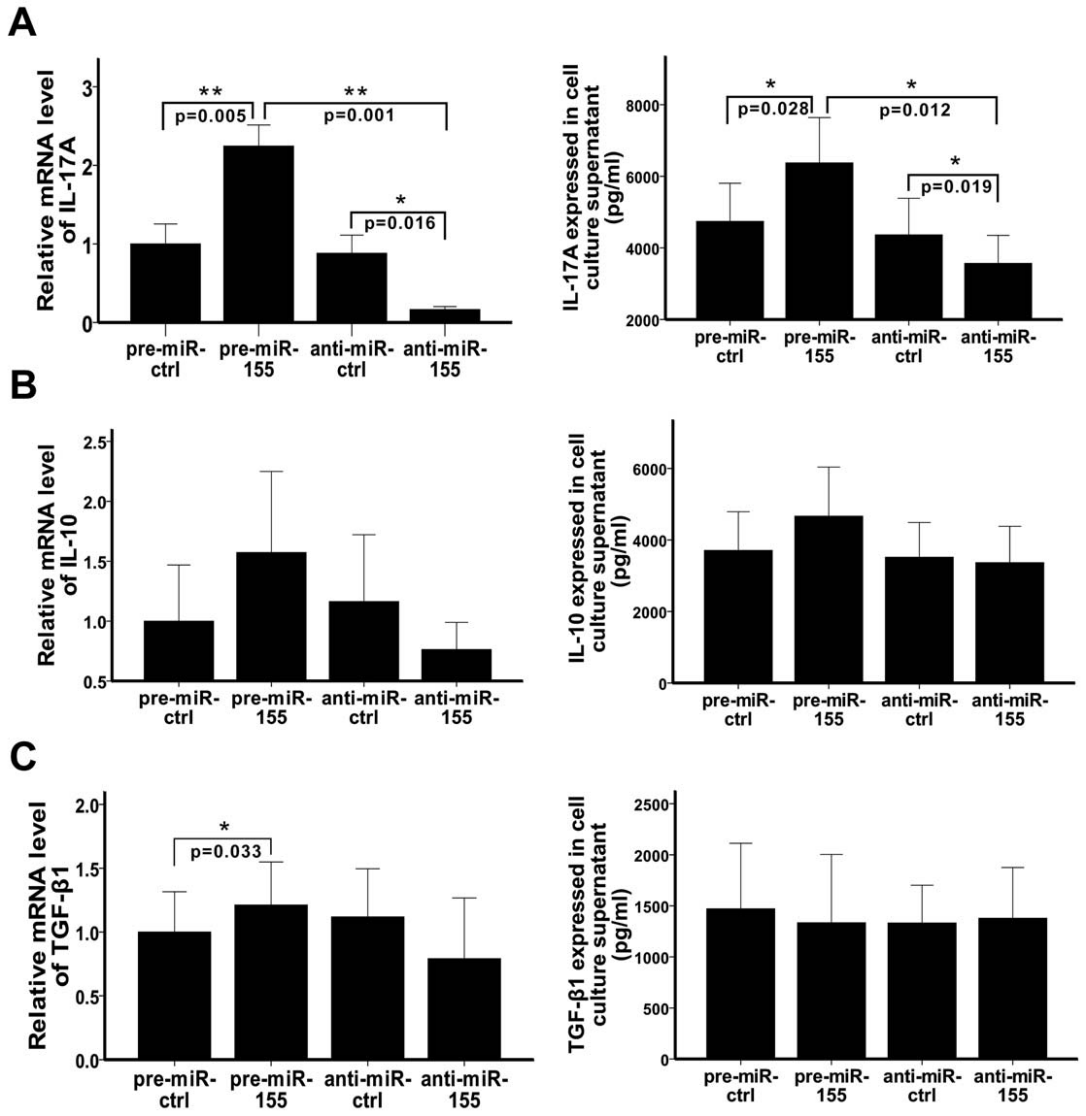
MiR-155 regulates the secreting of IL-17A, but not the secreting of IL-10 and TGF-β1. Pre-miR-ctrl, pre-miR-155, anti-miR-ctrl, and anti-miR-155 were transfected into CD4^+^ T cells, which were then activated and polarized. A–C. The mRNA levels of IL-17A (A), IL-10 (B) and TGF-β1 (C) were detected by RT-PCR 3 days after transfection and the collective results are shown in the left figures, respectively. While the levels of these cytokines in cell culture supernatant were detected by ELISAs 4 days after transfection and the collective results are shown in the right figures, respectively. All results are shown as mean ± SD. Data represent three independent experiments. *p<0.05, **p<0.01.

Th cell subsets play different roles mainly by elaborating distinct sets of cytokines. Th17 cell enhances inflammation by secreting IL-17A. Treg cell suppresses inflammation by secreting IL-10 and TGF-β1. To evaluate the influence of miR-155 on the function of Th17 and Treg cells, we detected the mRNA levels of IL-17A, IL-10, and TGF-β1 in CD4^+^ T cells as well as these cytokines secreted in cell culture supernatant. The results showed that both the mRNA level of IL-17A and IL-17A secreted in cell culture supernatant were significantly up-regulated by pre-miR-155 and were significantly down-regulated by anti-miR-155 (Fig. 2A). However, we did not observe statistically significant differences in the expression of IL-10 mRNA as well as IL-10 and TGF-β1 secreted in cell culture supernatant among the groups, although the mRNA level of TGF-β1 was slightly up-regulated by pre-miR-155 (Fig. 2B, 2C). As gene encoded protein mediates the biological effect, but not gene itself, so our results suggested that the function of TGF-β1 was not regulated by miR-155. Elevated mRNA level of TGF-β1 observed with pre-miR-155 treatment might because of the up-regulated Treg cell number. Still, more studies are necessary to clarify this issue. On the whole, we conclude that the production of IL-17A is induced by miR-155, but the production of IL-10 and TGF-β1 are not.

### MiR-155 reacts through regulating JAK/STAT rather than TGF-β/SMAD signaling pathway in the process of Treg and Th17 cells differentiation

**Figure 3:**
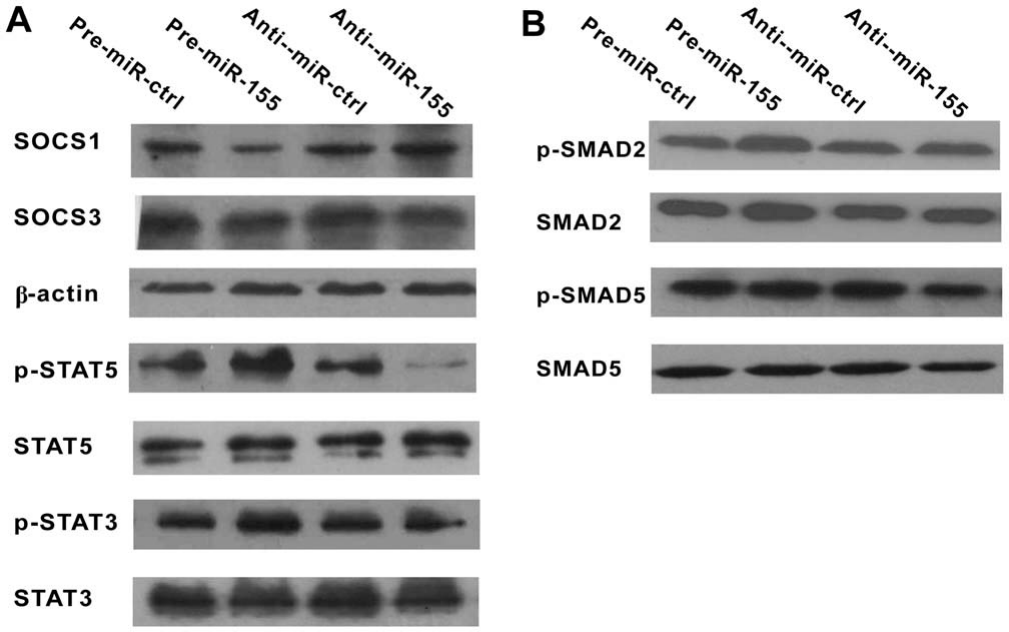
MiR-155 regulates JAK/STAT but not TGF-β/SMAD signaling pathway. 4 days after pre-miR-ctrl, pre-miR-155, anti-miR-ctrl, and anti-miR-155 were transfected, activated CD4^+^ T cells were collected. Whole-cell lysates were prepared and the transcripts involved in Treg and Th17 cells differentiation were detected by western blotting. A. First, protein lysates were immunoblotted with SOCS1 and SOCS3, and β-actin served as the internal reference. Then, protein lysates were immunoblotted with p-STAT5/p-STAT3 and the total proteins, STAT5/STAT3. B. Finally, protein lysates were immunoblotted with p-SMAD5/p-SMAD2 and the total proteins, SMAD5/SMAD2. Representative images from one of three independent experiments are shown.

Signaling pathways that contribute to Th cell differentiation had been well studied. Evidences showed that both STAT and mothers against decapentaplegic homolog (SMAD) family protein phosphorylations contribute to Treg and Th17 cells differentiation. Whereas SOCS1 inhibits both Treg and Th17 cells differentiation. In present study, we detected whether these transcripts were regulated by miR-155 in the process of Th cell activation and differentiation. The results showed that the expression of SOCS1 protein was decreased and signal transducer and activator of transcription (STAT)5 and STAT3 phosphorylations were increased by pre-miR-155, with no significant changes were found in the levels of β-actin and total STAT5 and STAT3 proteins, and opposite results were found when anti-miR-155 was transfected (Fig. 3A). SOCS3 is another important negative regulator of STAT signaling pathway, especially STAT3 [16]. But it was not regulated by miR-155 (Fig. 3A). Moreover, there were no statistically significant changes in SMAD2 and SMAD5 phosphorylations as well as the expression of the total proteins between the groups (Fig. 3B). So, the results reveal that miR-155 regulates STAT but not SMAD family protein phosphorylations during Treg and Th17 cells differentiation.

### SOCS1-TP^miR-155^ inhibits miR-155-mediated enhancement of Treg and Th17 cells differentiation and Th17 cell function

**Figure 4:**
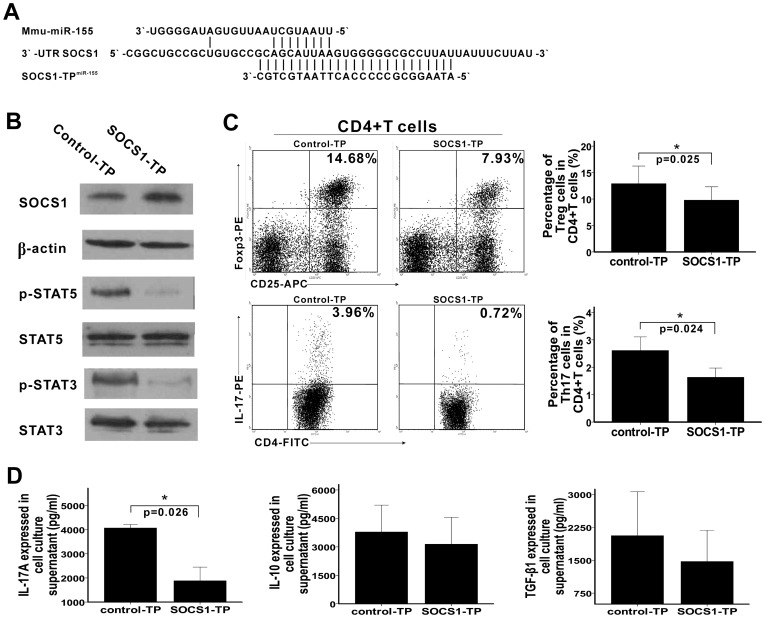
SOCS1-TP^miR-155^ inhibits the function of miR-155 during Treg and Th17 cells differentiation. A. Sequences of mmu-miR-155, 3′-UTR SOCS1, and SOCS1-TP^miR-155^ are shown in the schematic. As shown, seed sequences of mmu-miR-155 complementary to 3′-UTR SOCS1, whereas sequences of SOCS1-TP^miR-155^ completely complementary to 3′-UTR SOCS1 and over-lapped the binding site of miR-155 in the 3′ UTR of SOCS1. B. The percentages of Treg and Th17 were determined by flow cytometry 4 days after SOCS1-TP^miR-155^ and control-TP^miR-155^ (shown in the figures as SOCS1-TP and control-TP, respectively) were transfected. Treg and Th17 cells were gated with CD4^+^CD25^+^Foxp3^+^ and CD4^+^IL-17^+^, respectively. Typical FACS pictures from a single case are shown in the left. The collective results of three independent experiments are shown in the right as mean ± SD. C. 4 days after SOCS1-TP^miR-155^ and control-TP^miR-155^ were transfected, the levels of IL-17A, IL-10, and TGF-β1 in cell culture supernatant were quantified by ELISAs and the collective results are shown as mean ± SD. D. 4 days after SOCS1-TP^miR-155^ and control-TP^miR-155^ were transfected, the levels of the transcripts involved in Treg and Th17 cells differentiation were also detected by western blotting. Data represent three independent experiments. *p<0.05, **p<0.01.

Three transcripts, including SOCS1, SMAD2, and SMAD5, had been reported to be targets of miR-155. But we found that SOCS1 was the only one that was repressed by miR-155 in CD4^+^ T cells. To confirm this, target protector (TP) was used. As shown in Fig. 4A, SOCS1-TP^miR-155^ interferes miR-155-SOCS1 interaction by binding to the binding site of miR-155 in the 3′-untranslated region (UTR) of SOCS1, without interfering miR-155 interaction with other target mRNAs. So, when SOCS1-TP^miR-155^ was transfected, a dramatic increase of SOCS1 protein was observed, along with a corresponding decrease of STAT5 and STAT3 phosphorylations (Fig. 4B). Furthermore, the percentages of Treg and Th17 cells in CD4^+^ T cells as well as the expression of IL-17A in cell culture supernatant were decreased when SOCS1-TP^miR-155^ was transfected (Fig. 4C, 4D). However, we did not observe statistically significant changes in the expression of IL-10 and TGF-β1 in cell culture supernatant between control-TP^miR-155^ and SOCS1-TP^miR-155^ groups (Fig. 4D). Thus, SOCS1 is the direct target of miR-155 that involved in Treg and Th17 cells differentiation and Th17 cell function.

### MiR-155 regulates the expression of T-bet/GATA-3 and IFN-γ/IL-4 in the development of Th1/Th2 cells

**Figure 5:**
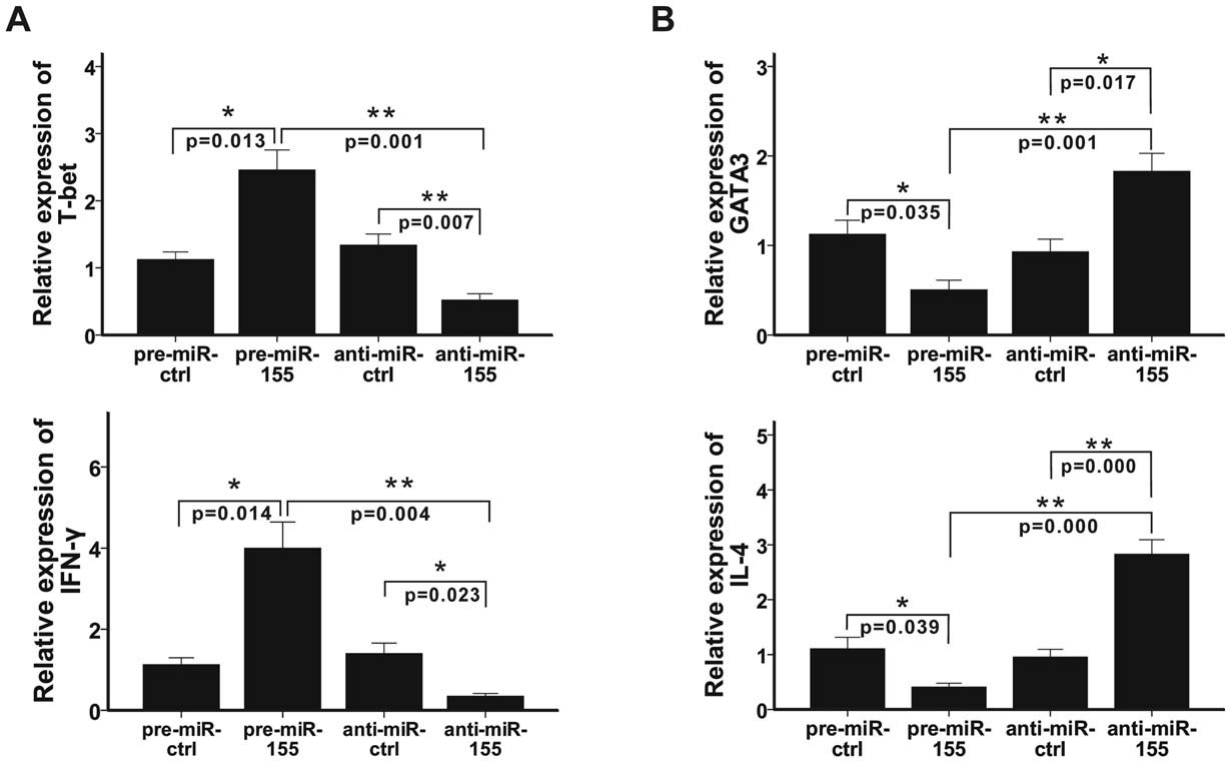
MiR-155 positively regulates the mRNA levels of T-bet and IFN-γ and negatively regulates the mRNA levels of GATA-3 and IL-4. Pre-miR-ctrl, pre-miR-155, anti-miR-ctrl, and anti-miR-155 were transfected into CD4^+^ T cells, which were then activated by anti-CD3 and anti-CD28. A. The mRNA levels of T-bet and IFN-γ were detected by RT-PCR 3 days after transfection and the collective results are shown, respectively. B. The mRNA levels of GATA3 and IL-4 were detected by RT-PCR 3 days after transfection and the collective results are shown, respectively. All results are shown as mean ± SD. Data represent more than three independent experiments. *p<0.05, **p<0.01.

Th1 and Th2 are important subsets of CD4^+^ T cells. Both Thai et al. [9] and Rodriguez et al. [10] had demonstrated that miR-155 knockout mice showed enhanced Th2 and diminished Th1 differentiation. Here, we detected the mRNA levels of the specific transcriptional regulators and the key functional cytokines of Th1 and Th2 cells when miR-155 was up-regulated and inhibited by pre-miR-155 and anti-miR-155, respectively. As shown in Fig. 5A, the mRNA levels of T-box expressed in T cells (T-bet) and IFN-γ were up-regulated by pre-miR-155 and down-regulated by anti-miR-155. In the contrast, the mRNA levels of GATA-binding protein-3 (GATA-3) and IL-4 were down-regulated by pre-miR-155 and up-regulated by anti-miR-155 (Fig. 5B). These results show that miR-155 induces the differentiation and function of Th1 cell and inhibits the differentiation and function of Th2 cell.

## Discussion

We have shown here that miR-155 is a critical player in driving Treg/Th17 cells differentiation and enhancing Th17 cell function by directly inhibiting SOCS1. SOCS1 is a negative regulator of Janus kinase (JAK)/STAT signaling pathway. Recently, the effect of SOCS1 on the development of Treg/Th17 cells has been studied. Lu et al. [14] and Zhan et al. [17] found that T-cell-specific deletion of SOCS1 resulted in an increase in the proportion and absolute numbers of Treg cells in the thymus. But it was not found that SOCS1 negatively regulated the suppressive function of Treg cells. The role of SOCS1 in Th17 cell differentiation and function was clarified by Johnson and his group by characterizing a mimetic of SOCS1, namely novel tyrosine kinase inhibitor peptide (Tkip). They found that Tkip blocked IL-6-induced activation of STAT3, inhibited the development of Th17 and the production of IL-17A [18]–[20]. As a result, the development of EAE in both acute and chronic phases was prevented by Tkip [21]. So, the differentiation of Treg and Th17 cells and the production of IL-17A were increased when the expression of SOCS1 was inhibited by miR-155.

Treg and Th17 cells are induced from uncommitted CD4^+^ T cells by different cytokine-driven signaling pathways. IL-6/STAT3 is indispensable for Th17 cells differentiation, and inhibits Treg cells [22]. Conditional deletion of STAT3 in CD4^+^ T cells impairs Th17 differentiation, IL-17A production, and limits EAE development [23]. Conversely, IL-2/STAT5 is essential for maintenance of homeostasis and competitive fitness of Treg cells, and suppresses Th17 differentiation [22], [24]. However, Fontenot et al. [25] found that the function of IL-2^−/−^ and IL-2rα^−/−^ Treg cells was equivalent to that of wild-type Treg cells, which indicated that IL-2/STAT5 signaling pathway was dispensable for Treg cell function. In the present study, we found that miR-155 positively regulated STAT5 and STAT3 phosphorylations. This must because miR-155 blocked the SOCS1-mediated inhibitory effect on STAT5 and STAT3 phosphorylations [25]–[27]. TGF-β1 is essential for both Treg and Th17 cells differentiation. It induces Foxp3 expression and promotes Treg cell function by activating SMAD5 signaling pathway [28]. It also works together with IL-6 to induce Th17 differentiation by activating SMAD2 signaling pathway [29]. Both SMAD5 and SMAD2 were validated to be targets of miR-155 in different papers. Louafi et al. [30] demonstrated that miR-155 modulated the response of macrophage to TGF-β1 by targeting SMAD2. While SMAD5 was found to be directly inhibited by miR-155 in B cell lymphoma cells [31]. But, in our study, neither the phosphorylations of SMAD5/SMAD2 nor the expression of the total proteins were regulated by miR-155 in CD4^+^ T cells, although phosphorylation of SMAD2 and the total proteins were down-regulated in RAW264.7 cells when miR-155 was over-expressed (data are not shown). This might because miR-155 prone to inhibit different transcripts in a cell-specific manner and SMAD2 was inhibited by miR-155 in macrophage and macrophage cell lines but not in T lymphocytes. Using SOCS1-TP^miR-155^, we confirmed that SOCS1 is the specific target of miR-155 that involved in inducing Treg and Th 17 differentiation and Th17 cell function, as what we found when SOCS1-TP^miR-155^ was transfected resembled those when anti-miR-155 was transfected.

Our study showed that the secreting of IL-17A by Th17 cells was positively regulated by miR-155, which was in accordance with what O'Connell et al. [15] and Murugaiyan et al. [32] found. However, no significant differences were found in the production of IL-10 and TGF-β1 by Treg cells between the groups. The functional imbalance of Treg/Th17 cells induced by miR-155 might contribute to the imbalance of the activation of JAK/STAT and TGF-β/SMAD signaling pathways induced by miR-155. STAT3 directly controls the expression of RORγt and IL-17A [33]. TGF-β/SMAD5 signaling pathway is critical for the function of Treg cells [28]. However, addition of IL-6/STAT3 to TGF-β/SMAD5 signaling pathway inhibits the function of Treg cells and induces the function of Th17 cells [29], [34]. Whereas IL-2/STAT5 is dispensable for Treg cell function. As miR-155 positively regulated IL-6/STAT3 and IL-2/STAT5, but not TGF-β/SMAD signaling pathway. So, the secreting of IL-17A was induced by miR-155, but IL-10 and TGF-β1 were not.

Th cell differentiation is regulated by a complex cytokine network. Th17 cells are induced from naive CD4^+^ T cells mainly by IL-6 and low concentrations of TGF-β1. But these cells are also regulated by cytokines secreted by most other Th cell subsets, including IL-4, interferon (IFN)-γ, IL-10 and at high concentrations, TGF-β1 [35]. In our study, miR-155 enhanced the differentiation and function of Th17 cells. On one hand, it is because miR-155 activated IL-6/STAT3 signaling pathway, which is pivotal for Th17 cells development and IL-17A production. On the other hand, it is because miR-155 impaired the inhibitory actions of IL-10 and TGF-β1 on the secreting of IL-17A by Th17 cells.

Besides Treg and Th17 cells, SOCS1 also regulates Th1 and Th2 cells differentiation and function. As a negative regulator, SOCS1 inhibits both STAT family proteins which regulate Th1 and Th2 differentiation. But high amount of SOCS1 inhibits Th1 cell differentiation, while promoting Th2 cell induction [36]. So, it is possible that miR-155 modulates Th1/Th2 differentiation by directly inhibiting SOCS1. Moreover, by targeting SOCS1, miR-155 also regulates functions of macrophages and DCs [37], [38]. So, miR-155 may regulate both innate and adaptive immune responses by targeting SOCS1. Additionally, altered expression of SOCS1 contributes to the progression of chronic inflammatory diseases, such as rheumatoid arthritis, systemic lupus erythematosus, and renal injury [39]–[41]. It was also involved in modulating of inflammatory processes within the atherosclerotic vascular wall. Ortiz-Muñoz et al. [42] demonstrated that over-expressed SOCS1 in vascular smooth muscle cells and macrophages inhibited IFN-γ/STAT1 signaling pathway, which has been found play important roles in the development of atherosclerosis [43].

**Figure 6:**
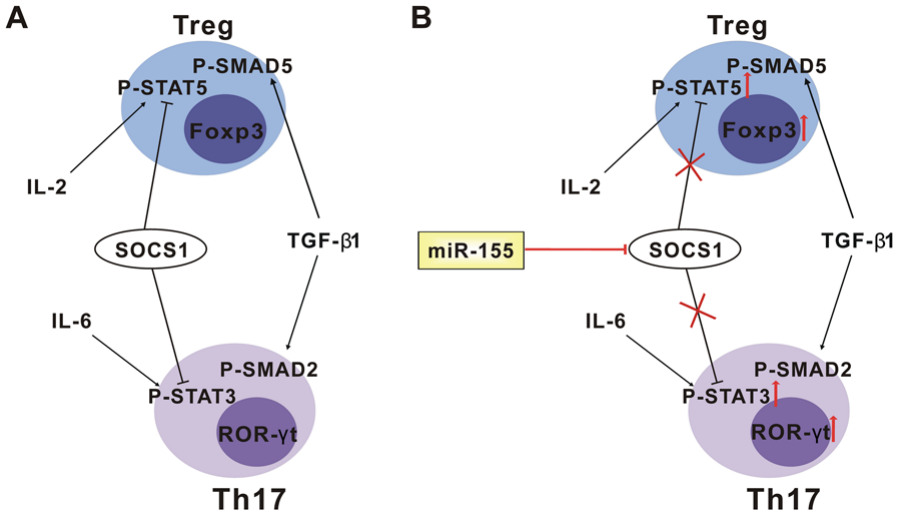
The mechanism of how miR-155 regulates Treg/Th17 cells differentiation and Th17 cell function. A. Signaling pathways that are involved in regulating Treg and Th17 cells differentiation and function. IL-2 and TGF-β1 induce the differentiation of Treg cells by enhancing the expression of phospho (p)-STAT5 and p-SMAD5, respectively. While IL-6 and TGF-β1 induce the differentiation of Th17 cells by enhancing the expression of p-STAT3 and p-SMAD2, respectively. SOCS1 negatively regulates both Treg and Th17 cells by inhibition the expression of p-STAT5 and p-STAT3, respectively. B. The mechanism of how miR-155 regulates Treg/Th17 cells differentiation and Th17 cell function. MiR-155 directly targets SOCS1, which inhibits the negative regulation of p-STAT5 and p-STAT3 by SOCS1 and induces Treg/Th17 cells differentiation, Foxp3/RORγt expression, and Th17 cell function.

In conclusion, we present evidence that miR-155 inhibits the expression of SOCS1 in activated CD4^+^ T cells, which, at least in part, contributes to the activation of IL-2/STAT5 and IL-6/STAT3 signaling pathways and the induction of Treg/Th17 cells differentiation and Th17 function (as shown in Fig. 6). We presume that miR-155 may be involved in modulating inflammatory diseases at least in part by regulating SOCS1. These may offer a new perspective on the study about miRNA-155 and inflammatory diseases. However, miR-155 is a typical multi-functional miRNA [44]. It also regulates the function of antigen-presenting cells (APCs), which indirectly modulates T lymphocytes development [10], [15]. Whereas we did this study in purified CD4^+^ T cells *in vitro*. So, more studies are necessary to clarify the specific roles of miR-155 in different inflammatory processes *in vivo*.

## Materials and Methods

### Ethics Statement

The study was carried out in strict accordance with the Guidelines For the Care and Use of Laboratory Animals (Science & Technology Department of Huibei Province, PR China, 2005). The protocol was approved by Animal Care and Use Committee of Hubei Province (Permit Number: 00017314). BALB/c mice were housed under specific pathogen-free (SPF) conditions with 12∶12 light∶dark cycle and 22±2°C and 60±5% humidity. Sterile water and chow were available ad libitum. They were maintained under these conditions until experiment protocols.

### Purification of CD4^+^ T cells

**Figure 7:**
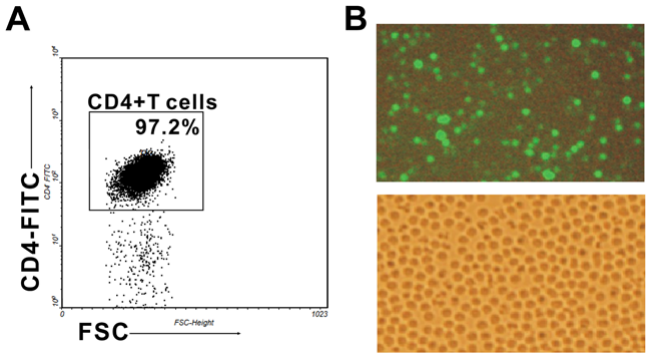
The purity of CD4^+^ T cells and the GFP-transfection efficiency in CD4^+^ T cells. A. CD4^+^ T cells were purified by magnetic cell sorting (MACS). The purity of CD4^+^ T cells was determined by flow cytometry and a typical FACS picture is shown. B. 2.5 µg pmaxGFP® Vector was transfected into 1×10^7^ CD4^+^ T cells by necleofection. The GFP-transfection efficiency was detected by fluorescence microscopy 8 h later and a typical 20× image (top) is shown. Same slide of CD4^+^ T cells was detected sequentially by light microscopy and the 20× image (bottom) is also shown. Both the purity and the transfection efficiency were checked in every independent experiment.

Splenic mononuclear cells were obtained from 6–8 week-old BALB/c mice by Ficoll-Hypaque density gradient centrifugation. Then, using CD4^+^ T Cell Isolation Kit II (Miltenyi Biotec, Auburn, CA, USA), naive CD4^+^ T cells were isolated by depletion of non-CD4^+^ T cells, including CD8a, CD11b, CD11c, CD19, CD45R, CD49b, CD105, Anti-MHC class II, and Ter-119 positive cells. The purity of CD4^+^ T cells was >95% (Fig. 7A)

### Nucleofection

Nucleofection was performed with Mouse T Cell Nucleofector® Kit and Nucleofector device (Amaxa, Koelin, Germany). First, 1×10^7^ naive CD4^+^ T cells were resuspended in 100 µl nucleofector® solution. 2.5 µg pmaxGFP® Vector or 100 pmol oligonucleotides (including pre-miR-155, pre-miR-ctrl, anti-miR-155, anti-miR-ctrl, SOCS1-TP^miR-155^, and control-TP^miR-155^) were added into the solution and mixed gently. Then the mixtures were gently transferred to electroporation cuvettes and placed in the Nucleofector device. Cells were nucleofected in the X-01 program. Finally, transfected cells were transferred to a 12-well plate with 1.5 ml prepared Mouse T Cell Nucleofector® Medium in each plate and incubated in a humidified 37°C/5% CO_2_ incubator until analysis. The transfection efficiency, which was evaluated by monitoring green fluorescent protein (GFP) expression under fluorescence microscope 8 h after transfection, is around 50% (Fig. 7B). In addition, pre-miR-155 and anti-miR-155 from Ambion (Austin, TX, USA) were used to over-express and inhibit of miR-155, with pre-miR-ctrl and anti-miR-ctrl as the matched controls, respectively. SOCS1-TP^miR-155^ from Gene Tools, LLC (Corvallis, OR, USA) was used to interfere miR-155 and suppressors of cytokine signaling1 (SOCS1) interaction, with control-TP^miR-155^ as the matched control.

### CD4^+^ T cell activation and polarization

4 h after nucleofection, CD4^+^ T cells were activated by 5 µg/ml plate-bound anti-CD3 and 2 µg/ml soluble anti-CD28. For propagation under Treg condition, 100 u/ml rIL-2, 10 ng/ml rTGF-β1, 10 µg/ml anti-IFN-γ, and 10 µg/ml anti-IL-4 were provided. For propagation under Th17 condition, 2.5 ng/ml rTGF-β1, 30 ng/ml rIL-6, 10 µg/ml anti-IFN-γ, and 10 µg/ml anti-IL-4 were provided. All antibodies used were purchased from ebioscience (San Diego, CA, USA). All cytokines used were purchased from Peprotech (Rocky Hill, NJ).

### Flow cytometry analysis

4 days after transfection and activation, cells were collected and used for detecting Treg and Th17 cells differentiation. For detecting Th17 cell differentiation, cells were stimulated for 4 h with 25 ng/ml phorbol myristate acetate (PMA) and 1 µg/ml ionomycin in the presence of 2 µM monensin in the last 2 h. Cells were collected and stained with FITC-labeled anti-mouse CD4 for 30 min at 4°C. Then, cells were fixed and permeabilized according to the manufacturer's protocol. Next, cells were incubated with PE-labeled anti-mouse IL-17A in the dark for 20 min. For detecting Treg cells differentiation, cells were stained with FITC-labeled anti-mouse CD4 and APC-labeled anti-mouse CD25 for 30 min at 4°C. After fixation and permeabilization, PE-labeled anti-mouse Foxp3 were provided. Finally, stained cells were resuspended in 200 µl washing buffer and analyzed by FACS Calibur Flow Cytometer (BD Biosciences, San Jose, CA, USA). All reagents used were purchased from eBioscience (San Diego, CA, USA).

### RT-PCR

Total RNA was extracted from CD4^+^ T cells with TRIzol reagents (invitrogen, USA) following the manufacturer's protocol. The purity of RNA was acceptable when the OD at 260 and 280 nm (A_260/280_) was between 1.8 and 2.0. RT-PCR was performed according to the protocol of One Step SYBR® PrimeScript™ RT-PCR Kit II (TaKaRa, Shiga, Japan) and 1 µg purified RNA was provided to 20 µl volume. Each target was measured in triplicate and normalized to the level of β-action. The PCR primers used in our study had been described previously [45], [46].

### ELISA

Cell culture supernatant was collected 4 days after CD4^+^ T cells were transfected and activated. The levels of IL-17A, IL-10, and TGF-β1 in the cell culture supernatant were determined with ELISA kits (Bender MedSystems, Vienna, Austria) following the manufacturer's introduction. The minimal detectable concentrations were 0.5 pg/ml for IL-17A, 5 pg/ml for IL-10, and 9 pg/ml for TGF-β1. To determine the levels of IL-17A, IL-10, and TGF-β1 precisely, samples were diluted by Sample Diluent for 10, 6, and 2 times, respectively. The standard curves were prepared using 1∶2 serial diluted IL-17A, IL-10, and TGF-β1 standards, respectively. All samples were measured in triplicate. Interassay and intraassay coefficients of variation for the ELISAs were <10% and <5%, respectively.

### Western blotting

Protein extracts were prepared in lysis buffer with proteinase inhibitor as well as phosphatase inhibitor. Whole-cell lysates (50 µg) were separated in 9% or 12% sodium dodecyl sulfate-polyacrylamide gel electrophoresis (SDS-PAGE) gels and then electroblotted onto a nitrocellulose. After incubation for 1 h in 5% skim milk (w/v) diluted in Tris-buffered saline with Tween 20 (TBST) at room temperature, the membrane was incubated overnight at 4°C with a dilution between 1/500 and 1/1000 of primary antibodies. Then washed 3×15 min with TBST, incubated for 2 h with a 1∶2000 dilution of horseradish peroxidase-conjugated goat anti-rabbit or anti-mouse IgG secondary antibody for 2 h at room temperature. The membrane was washed 3×15 min with TBST again and the blots were developed by enhanced chemiluminescence (ECL kit; Amersham). The levels of phosphorylated proteins were normalized to their total protein levels and other proteins were normalized to the signal generated for β-actin. All reagents used were purchased from cell signaling technology (Beverly, MA, USA).

### Statistical analysis

All values were expressed as mean ± SD. Statistical significance was determined by performing ANOVAs for multiple comparisons and Student's t test for two comparisons. P values<0.05 were considered statistically significant and <0.01 were considered highly statistically significant.
